# Root inoculation with *Pseudomonas putida* KT2440 induces transcriptional and metabolic changes and systemic resistance in maize plants

**DOI:** 10.3389/fpls.2014.00719

**Published:** 2015-01-13

**Authors:** Chantal Planchamp, Gaetan Glauser, Brigitte Mauch-Mani

**Affiliations:** ^1^Laboratory of Molecular and Cell Biology, Institute of Biology, University of NeuchâtelNeuchâtel, Switzerland; ^2^Chemical Analytical Service of the Swiss Plant Science Web, University of NeuchâtelNeuchâtel, Switzerland

**Keywords:** maize, *Pseudomonas putida*, *Colletotrichum graminicola*, induced systemic resistance, secondary metabolites, beneficial microorganisms, root colonization, hormone signaling

## Abstract

*Pseudomonas putida* KT2440 (KT2440) rhizobacteria colonize a wide range of plants. They have been extensively studied for their capacity to adhere to maize seeds, to tolerate toxic secondary metabolites produced by maize roots and to be attracted by maize roots. However, the response of maize plants to KT2440 colonization has not been investigated yet. Maize roots were inoculated with KT2440 and the local (roots) and systemic (leaves) early plant responses were investigated. The colonization behavior of KT2440 following application to maize seedlings was investigated and transcriptional analysis of stress- and defense-related genes as well as metabolite profiling of local and systemic maize tissues of KT2440-inoculated were performed. The local and systemic responses differed and more pronounced changes were observed in roots compared to leaves. Early in the interaction roots responded via jasmonic acid- and abscisic acid-dependent signaling. Interestingly, during later steps, the salicylic acid pathway was suppressed. Metabolite profiling revealed the importance of plant phospholipids in KT2440-maize interactions. An additional important maize secondary metabolite, a form of benzoxazinone, was also found to be differently abundant in roots 3 days after KT2440 inoculation. However, the transcriptional and metabolic changes observed in bacterized plants early during the interaction were minor and became even less pronounced with time, indicating an accommodation state of the plant to the presence of KT2440. Since the maize plants reacted to the presence of KT2440 in the rhizosphere, we also investigated the ability of these bacteria to trigger induced systemic resistance (ISR) against the maize anthracnose fungus *Colletotrichum graminicola*. The observed resistance was expressed as strongly reduced leaf necrosis and fungal growth in infected bacterized plants compared to non-bacterized controls, showing the potential of KT2440 to act as resistance inducers.

## Introduction

Plants are regularly challenged by a wide variety of pathogenic microbes present in their direct environment. However, beneficial interactions between plants and microorganisms are also frequent in nature and known to improve plant nutrition and/or help plants overcome biotic or abiotic stresses. Plants therefore have to rapidly recognize the interacting partner and activate an adapted response. Interestingly, there is increasing evidence that plants react in a similar way toward pathogenic and non-pathogenic microbes: both are recognized based on microbe-associated molecular patterns (MAMP). Several MAMPs of beneficial microbes such as lipopolysaccharides, flagellin or secondary metabolites have been shown to activate MAMP-triggered immunity (MTI; Van Loon et al., 2008; Hermosa et al., 2012; Zamioudis and Pieterse, 2012). However, in order to establish a beneficial interaction involving an efficient bacterial colonization, there is a necessity to suppress MTI following recognition of the microbe (Gutjahr and Paszkowski, 2009; Zamioudis and Pieterse, 2012). Beneficial microbes are able to suppress the plant reaction via the release of effector molecules or by manipulating the plant hormonal balance. As described for some pathogens such as *Pseudomonas syringae* pv. *tomato* DC3000 (*Pst*) in Arabidopsis, beneficial microbes are also able to exploit plant hormone crosstalk to overcome MTI and to enhance their colonization capacity. Thus, root colonization by the PGPF (plant growth promoting fungus) *Piriformospora indica* was associated with jasmonic acid- (JA) dependent signaling during its biotrophic phase, followed by the involvement of gibberellic acid (GA) during the cell death associated phase (Jacobs et al., 2011). Colonization of rice roots by the mycorrhizal fungus *Glomus intraradices* was associated with local and systemic up-regulation of the JA-dependant pathway and the down-regulation of *PR* gene expression (Campos-Soriano et al., 2012). Plant growth promoting rhizobacteria (PGPR) may also produce phytohormones to attenuate plant responses via hormonal manipulations. Jasmonic acid signaling was for example up-regulated in wheat in response to *Pseudomonas fluorescens* Q8r1-96 (Okubara et al., 2010). The local transcriptional response of Arabidopsis roots upon *P. fluorescens* GM30 colonization showed an up-regulation of the abscisic acid- (ABA) and ethylene- (ET) dependent pathway (Weston et al., 2012). On the contrary, the interaction between *P. fluorescens* SS101 and Arabidopsis resulted in a SA-dependent response (Van de Mortel et al., 2012).

Several changes in plant physiology have been described to occur in a bacterial strain-plant cultivar dependent manner during beneficial interactions between plant and soil-borne microbes. Hence, the inoculation of maize with *Azospirillum* strains induced different changes of defense compounds depending on the bacterial strain and maize cultivar involved in the interaction (Walker et al., 2011). During beneficial interactions, as well as for pathogenic interactions, many changes occur in membrane lipids (Cowan, 2006). Plant primary metabolism is affected by beneficial interaction triggered by PGPR such as *Azotobacter* (Kumar et al., 2007) and *P. fluorescens*. Levels of the amino acids tryptophan and phenylalanine were altered in the shoots of Arabidopsis following root inoculation with *P. fluorescens* GM30 or Pf-5 (Weston et al., 2012). Secondary metabolites are important for plant defense but their presence also fluctuates in response to beneficial interactions. Benzoxazinones (BXD), such as 2,4-dihydroxy-7-methoxy-1,4-benzoxazin-3-one (DIMBOA), are well-known defense compounds of the *Poaceae* family that are efficient against herbivores, aphids and fungi (reviewed in Niemeyer, 2009; Glauser et al., 2011). Significant changes in BXD content were observed in maize plants upon mycorrhizal or rhizobacterial colonization. Whereas the presence of *Glomus mosseae* induced a systemic accumulation of DIMBOA (Song et al., 2011), interaction with *Azospirillum* caused a local decrease of DIMBOA, DIMBOA-glucoside (DIMBOA-Glc), and 2-hydroxy-4,7-dimethoxy-1,4-benzoxazin-3-one glucoside (HDMBOA-Glc; Walker et al., 2011). Other phytoalexins such as coumarins and flavonoids also quantitatively changed in plants that were in association with rhizobacteria (Dardanelli et al., 2010; Drogue et al., 2012; Van de Mortel et al., 2012). These effects on metabolite profiles suggest the establishment of complex responses during beneficial interactions.

*Pseudomonas putida* KT2440 (KT2440) are gram-negative rhizobacteria that have the capacity to adapt to various niches such as soil or polluted environments (Wu et al., 2010). These rhizobacteria are also able to colonize a wide range of plants and have been extensively studied for their capacity to adhere to maize seeds (Espinosa-Urgel and Ramos, 2004) and to regulate colonization via cell–cell communication (Fernández-Piñar et al., 2012). A detailed analysis of the genome of KT2440 revealed the presence of genes involved in pathways linked to plant growth promoting properties, such as the production of phytohormones (Wu et al., 2010). KT2440 show a specific ability to live in the maize rhizoplane: these bacteria are tolerant to DIMBOA, a biocidal compound released by maize plants into the rhizosphere, and they exploit this chemical as a chemotaxis signal (Neal et al., 2012). Despite extended studies on KT2440, the response of plant upon KT2440 colonization has not been investigated yet. In our study, maize plants were inoculated with KT2440 and the plant response to this rhizobacteria–plant interaction was investigated at the transcriptional and metabolomic levels. We found that KT2440 elicited a host plant response that could benefit the rhizobacterial colonization.

While herbivore-induced defenses and indirectly induced defenses of maize plants are well documented (Turlings et al., 1995; Rasmann et al., 2005; Ton et al., 2006; Chen, 2008; Erb et al., 2009), only few studies have reported on direct systemic induced resistance such as induced systemic resistance (ISR; Balint-Kurti and Johal, 2009; Balmer et al., 2012). The studied interactions between maize plants and beneficial microorganisms concern essentially growth promotion or improved tolerance against abiotic stresses (Nadeem et al., 2006, 2007), bioremediation, and direct antagonistic effect against soil-borne pathogens (Wicklow and Poling, 2009). ISR has been documented in maize in response to the root colonizing fungi *Trichoderma harzianum* (Harman et al., 2004) and *Trichoderma virens* (Djonovic et al., 2007). Only two studies have reported the elicitation of ISR in maize by PGPR. *Bacillus cereus* C1L induced maize resistance against *Cochliobolus heterostrophus*, the causal agent of southern corn leaf blight, via the release of dimethyl disulfide (Huang et al., 2012) and the *Pseudomonas aurantiaca* JD37 induced resistance against the same pathogenic fungus (Fang et al., 2012). Previous studies showed that KT2440 were able to induce systemic resistance in Arabidopsis against *Pst* (Matilla et al., 2010) and to prime maize defense response to a herbivore-mimicking treatment (wounding and JA treatment; Neal and Ton, 2013). However, there is no biological evidence for their capacity to induce maize resistance against biotic stresses. For these reasons we also examined the potential of KT2440 of promoting maize defense against a hemibiotrophic pathogen, *Colletotrichum graminicola*, the causal agent of maize anthracnose.

## Materials and methods

### Biological material

Prior to germination, maize seeds (var. Golden Jubilee, West Coast Seeds, Canada) were first rinsed 3–5 times in 70% ethanol, then sterilized in bleach 10% for 5 minutes and washed five times in sterile distilled water. Seeds were then placed for germination in humid rolled filter paper (Filterkrepp Papier braun, 100g m^−2^, Weber and Cie, 8157 Dielsdorf, Switzerland) for 2 days in a growth chamber (16 hours day at 26°C, 8 hours night at 22°C, 60% relative humidity and an irradiance of 400 μmol m^−2^ s^−1^). After 2 days, seedlings were transferred to a soil-free root observation system (SF-ROBS) as described (Planchamp et al., 2013). Seedlings were grown in the same conditions as for germination.

The rifampicin-resistant strain *P. putida* KT2440 (KT2440) stock was kept in glycerol 50% at −80°C until use. For each experiment, a bacterial culture was made from a fresh stock on Luria–Bertani (LB; Difco LB, Becton, Dickinson and Company, France) agar supplemented with 100 μg mL^−1^ of rifampicin. After 2–5 days of growth in the dark at 25°C, a single colony of KT2440 was picked and transferred to 100 mL of LB liquid medium supplemented with 100 μg mL^−1^ of rifampicin for an overnight culture at 28°C, under continuous shaking at 150 rpm. Seeds pre-germinated for 2 days were inoculated with a bacterial suspension, for bacterized plants, or sterile M9 minimal medium, for control (or mock-treated) plants, as previously described (Planchamp et al., 2013).

In order to assess the effect of bacterial root colonization over time, maize roots and leaves of nine plants (per treatment) were collected at different times (3, 5, and 10 days) after seedling inoculation with KT2440. As leaves were not enough developed 3 days after inoculation with KT2440, no leaf material was collected for this time point. The plant material was used to test the bacterial colonization over time and for gene expression and metabolite analysis. The material of two independent experiments was pooled together to minimize possible biological variation (representing 18 plants per treatment, per collection time point).

A GFP-expressing strain of the hemibiotrophic fungus, *C. graminicola* (Erb et al., 2011), the causal agent of corn anthracnose, was used for fungal infection tests. The fungus was maintained on potato dextrose agar (PDA; Difco PDA, Becton, Dickinson and Co., France) at 25°C, under continuous light (70 μmol m^−2^ s^−1^).

### Bacterial quantification

In order to test for bacterial presence in roots during plant growth, roots from six bacteria-inoculated or mock-inoculated seedlings were collected and quickly blotted dry 3, 5 and 10 days after bacterial or mock treatment. Hundred milligram of fresh weight per sample were separately ground in 600 μL of sterile MgSO_4_ 10 mM. Serial dilutions were plated on King's medium B supplemented with 100 μg of rifampicin and were incubated at 25°C in the dark for 18–20 hours. The colony-forming units (CFU) per gram of fresh root were then determined.

To test for a possible direct inhibitory effect of KT2440 against *C. graminicola*, at the end of each induced resistance assay, roots and shoots from treated and control plants were collected and tested for the presence of KT2440 as described above by grinding 100 or 50 mg of fresh root or leaf tissues, respectively.

### Gene expression analysis

The whole root system (except crown roots) and leaf material (second and third leaves) from six biological replicates (one replicate representing a pool of three plants) were flash-frozen after collection and separately ground with a mortar and a pestle taking care to keep the plant material frozen. RNA extraction, cDNA synthesis, qRT-PCR analysis, and relative gene expression calculation were then performed as described by Balmer et al. (2013). Target gene expression was calculated by comparison with two housekeeping gene: *Zm-GAPc* and *Zm-Actin*. Primers for qRT-PCR that were used in this study are listed in a supplementary file (Table S1). Relative gene expression was calculated following the Pfaffl model (Pfaffl, 2001) and a statistical analysis was performed by using REST 2009 (Qiagen).

### Metabolomic analysis and quantitative measurements of benzoxazinones

Material prepared for gene expression analysis was also used for metabolomic analysis and quantitative measurement of BXD. Metabolites were extracted from 100 mg of frozen ground tissue in a mixer mill using 1 mL of extraction solvent (methanol 70%, water 29.5%, formic acid 0.5%). Plant extracts were then analyzed either non-diluted for metabolite fingerprinting or diluted 10 times with the extraction solvent for BXD quantification. The quantification of BXD was performed according to Meihls et al. (2013). For metabolic fingerprinting, non-diluted extracts were analyzed using a UHPLC-QTOF-MS system (Waters) equipped with an electrospray (ESI) interface. An Acquity BEH C18 column (2.1 × 1.7 μm, Waters) was used for separation at a flow rate of 400 μL min^−1^. The mobile phase consisted of two solvents: solvent A (water and formic acid 0.05%) and solvent B (acetonitrile and formic acid 0.05%). The gradient program was employed as follows: 0–3.5 min 2–27.2% B, 3.5–8 min 27.2–100% B, holding at 100% B for 1.5 min, re-equilibration at 2% B for 1.5 min. The column temperature was maintained at 40°C and the injection volume was 3 μL. Detection was performed in ESI positive ion mode over an *m/z* range of 85–1200 Da with a scan time of 0.4 s. QTOF-MS settings were: capillary voltage at +2800 V, cone voltage at +25 V, source temperature at 120°C, desolvation temperature, and gas flow at 330°C and 800 L h^−1^, respectively. A 400 ng mL^−1^ solution of peptide leucine enkephalin was used as an internal reference for ensuring accurate mass measurements. MassLynx™ V.4.1. was used to control the system.

Data were processed as in Balmer et al. (2013). Interesting markers were tentatively identified as follows: molecular formulae were computed according to mass and spectral accuracies using the elemental composition calculator provided by MassLynx™. Proposed molecular formulae were then imported and verified into different metabolite databases such as the Dictionary of Natural Products, the KNapSAcK Core system and the Lipid Maps database (http://www.lipidmaps.org/data/structure/LMSDSearch.php?Mode=SetupTextOntologySearch) to verify if those could correspond to known compounds. The putative structures were then matched with the MS/MS fragments obtained at high collision energy using MassFragment™ (Waters).

### Induced resistance assays

Spores from a 3-week old *C. graminicola* culture were collected in MgSO_4_ (containing 0.01% of Silwet L-77; Lehle Seeds, USA) and the spore concentration was adjusted to 10^6^ spores mL^−1^. Twelve-day old maize plants (i.e., 10 days after bacterial or mock inoculation) were infected or mock-treated (*n* = 12 plants per treatment) as follows: 20 μL drops of spore suspension or MgSO_4_ were spread with a paintbrush on 2–4 spots on the surface of the second and the third maize leaves (var. Golden Jubilee). After inoculation, plants were kept at 25°C in the dark and high relative humidity (>90%) for 16 hours. After this incubation time, plants were returned to the growth chamber. Four days after infection, fungal colonization was quantified as described previously (Erb et al., 2011) by calculating a ratio between the fungus and the leaf on a predetermined area. Two independent experiments were pooled together (*n* = 24 plants).

### Statistical analysis

Statistical analysis of metabolites and gene expression analysis were performed as described above. The rest of the comparisons were accomplished using SigmaPlot 11.0. Depending on the data distribution, a Student *t*-test or a Mann–Whitney Rank Sum Test was used for comparison between two groups of data. The comparison of bacterial numbers in roots was achieved with a One-Way ANOVA on Rank, followed by a Tukey Test (*p* < 0.05).

## Results

### Bacterial root colonization during plant growth

In order to test root colonization by *P. putida* KT2440 during maize growth, the number of colony-forming units of these bacteria extracted from inoculated roots was assessed at 3, 5, and 10 days post bacterial inoculation (dpi). In mock-inoculated roots, no bacteria were found. In bacterized roots, there were no statistical differences between 3 dpi (2 × 10^7^ CFU g^−1^ of fresh roots) and 5 dpi (8 × 10^6^ CFU g^−1^ of fresh roots; One-Way ANOVA on Rank, followed by a Tukey Test, *p* > 0.05). In comparison to 3 and 5 dpi, there were fewer bacteria present in roots at 10 dpi (1 × 10^5^ CFU g^−1^ of fresh roots; One-Way ANOVA on Rank, *p* < 0.05).

### Local and systemic changes in plant gene expression upon KT2440 inoculation

**Figure 1:**
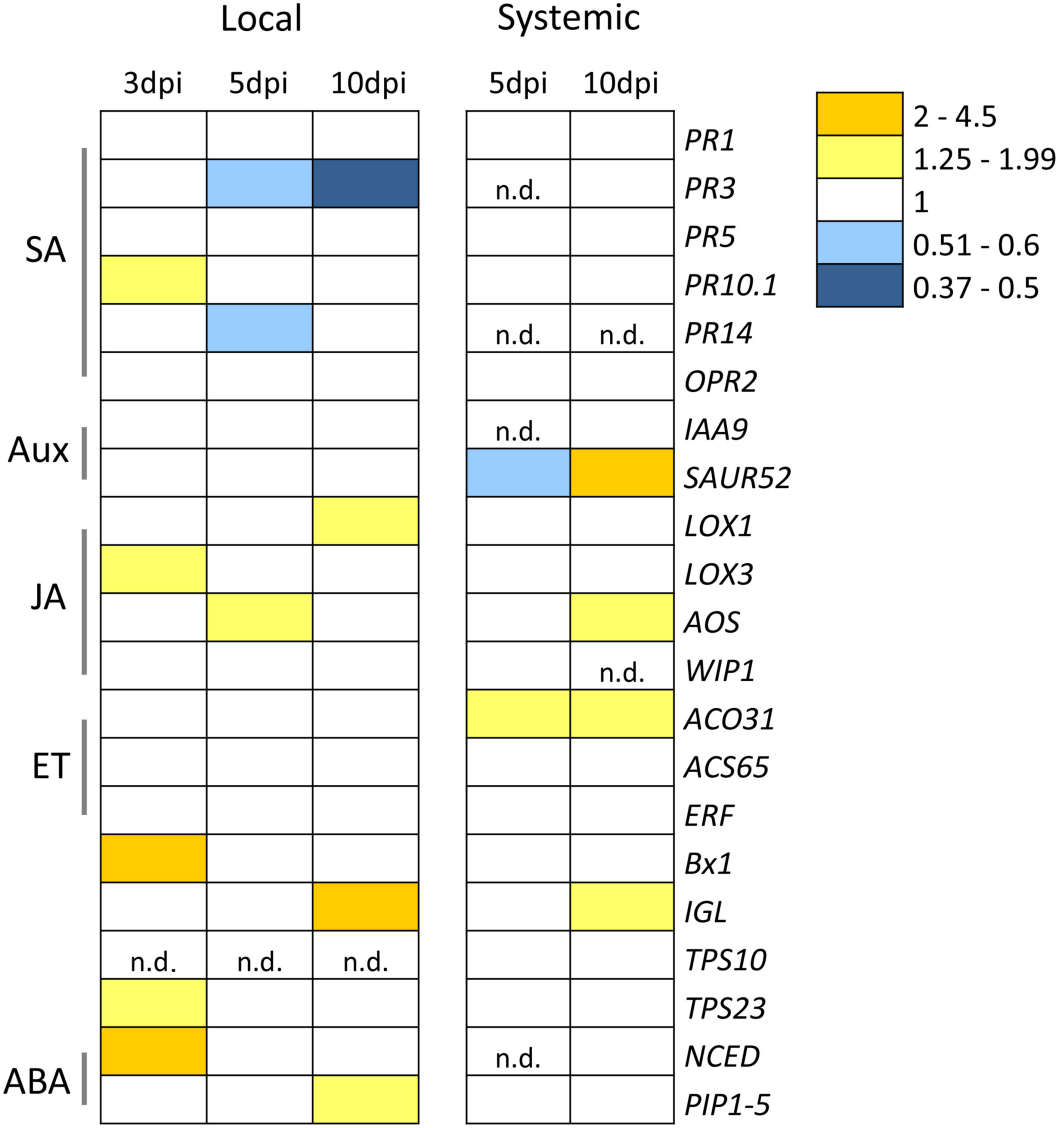
**Local (roots) and systemic (leaves) relative gene expression profile of maize plants (var. Jubilee) upon KT2440 root inoculation**. Plant material was collected 3, 5, and 10 days post inoculation (dpi). Results show the qRT-PCR analysis of the plant material of two independent experiments pooled together (*n* = 6 replicates, each replicate representing a pool of three plants). Gene expression is indicated as fold induction compared to non-bacterized plants. Yellow color indicates statistically significant gene up-regulation and blue color refers to down-regulated genes. White color shows no statistical differences in gene expression. n.d., not detected (i.e., either incomplete reaction or below detection threshold). SA, salicylic acid; Aux, auxin; JA, jasmonic acid; ET, ethylene; BXD, benzoxazinoid; ABA, abscisic acid.

The plant response upon KT2440 inoculation during maize growth was tested at the transcriptional level locally (in roots) as well as systemically (in leaves) by qRT-PCR. The relative expression of 21 stress-related genes of maize (Erb et al., 2009; Balmer et al., 2013) was calculated by comparing control and bacteria-inoculated plants at different time points (3, 5, and 10 dpi). Only few changes were observed (Figure 1 and Table S2). The local and systemic responses were different and more transcriptional changes were observed in roots compared to leaves. The majority of down-regulated genes in roots were linked to the SA signaling pathway (genes coding for the endochitinase *PR3* and the ribonuclease *PR14*) and observed from 5 dpi on. Earlier, at 3 dpi, there was an up-regulation of *PR10.1*, a SA-related pathway gene. Genes involved in indole (a volatile involved in defense) production as well as BXD precursors, *Bx1* and *IGL* (Ahmad et al., 2011), were up-regulated in roots at 3 and 10 dpi, respectively. *IGL* was also up-regulated in leaves at 10 dpi. *TPS23*, a gene coding for the synthesis of the volatile (*E*)-β-caryophyllene, was up-regulated in roots at 3 dpi. Abscisic acid (ABA)-related genes (*NCED* and *PIP1-5*) were up-regulated in roots at 3 and 10 dpi and remained unchanged in leaves. *NCED* was only marginally 3.96 fold up-regulated in roots of KT2440-inoculated plants 10 dpi (*p* = 0.07). JA-pathway related genes showed an overall slight induction in roots but only at 10 dpi in leaves. Interestingly, genes related to the auxin and ethylene pathway were only activated in leaves but not in roots. *SAUR52*, an auxin responsive gene, was down-regulated in leaves at 5 dpi, but up-regulated later in plant growth (10 dpi).

### Local and systemic changes in plant metabolites upon KT2440 inoculation

To gain more insight into KT2440-related changes in maize plants, metabolite changes were also analyzed. The plant responses upon KT2440 inoculation were determined at the metabolomic level using UHPLC-QTOF-MS. Secondary metabolites were measured in root and leaf tissues at different time points after bacterial inoculation (3, 5, and 10 days) in order to obtain a general view of metabolic changes and identify compounds that could serve as markers in the beneficial interaction between maize and KT2440.

**Figure 2:**
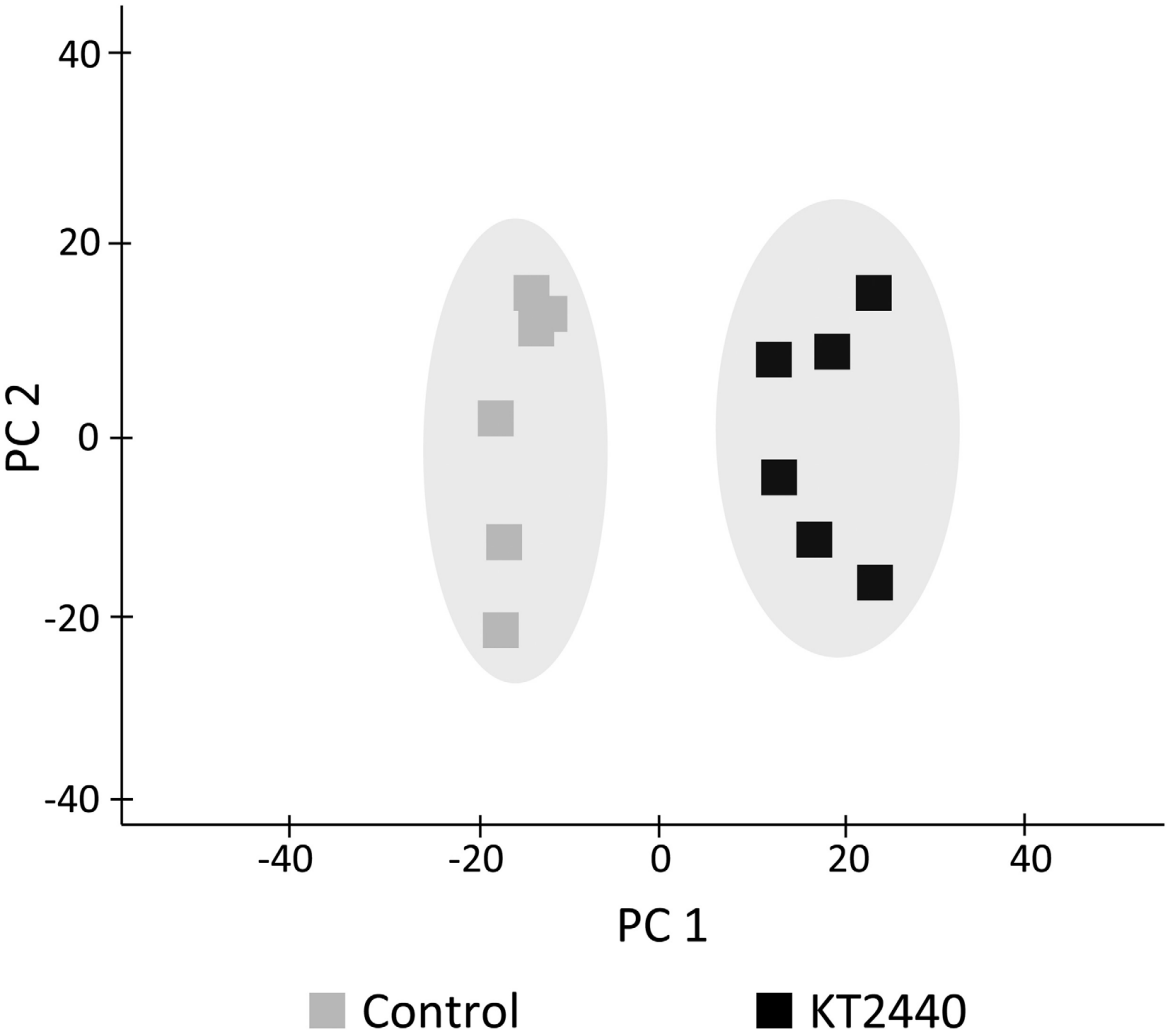
**Principal component analysis (PCA) score plot of metabolite fingerprinting of maize roots (var. Jubilee) 3 days after root inoculation with KT2440 (*n* = 6 replicates, each replicate representing a pool of 3 plants)**. Gray squares represent non-bacterized plants and black squares represent bacterized plants.

A principal component analysis (PCA) was performed to test possible separations between treatments within time points. PCA of metabolic fingerprints of roots at 3 dpi separated control from KT2440-inoculated plants along the first axis (PC1; Figure 2). This result indicates distinct metabolomic profiles between treatments.

**Figure 3:**
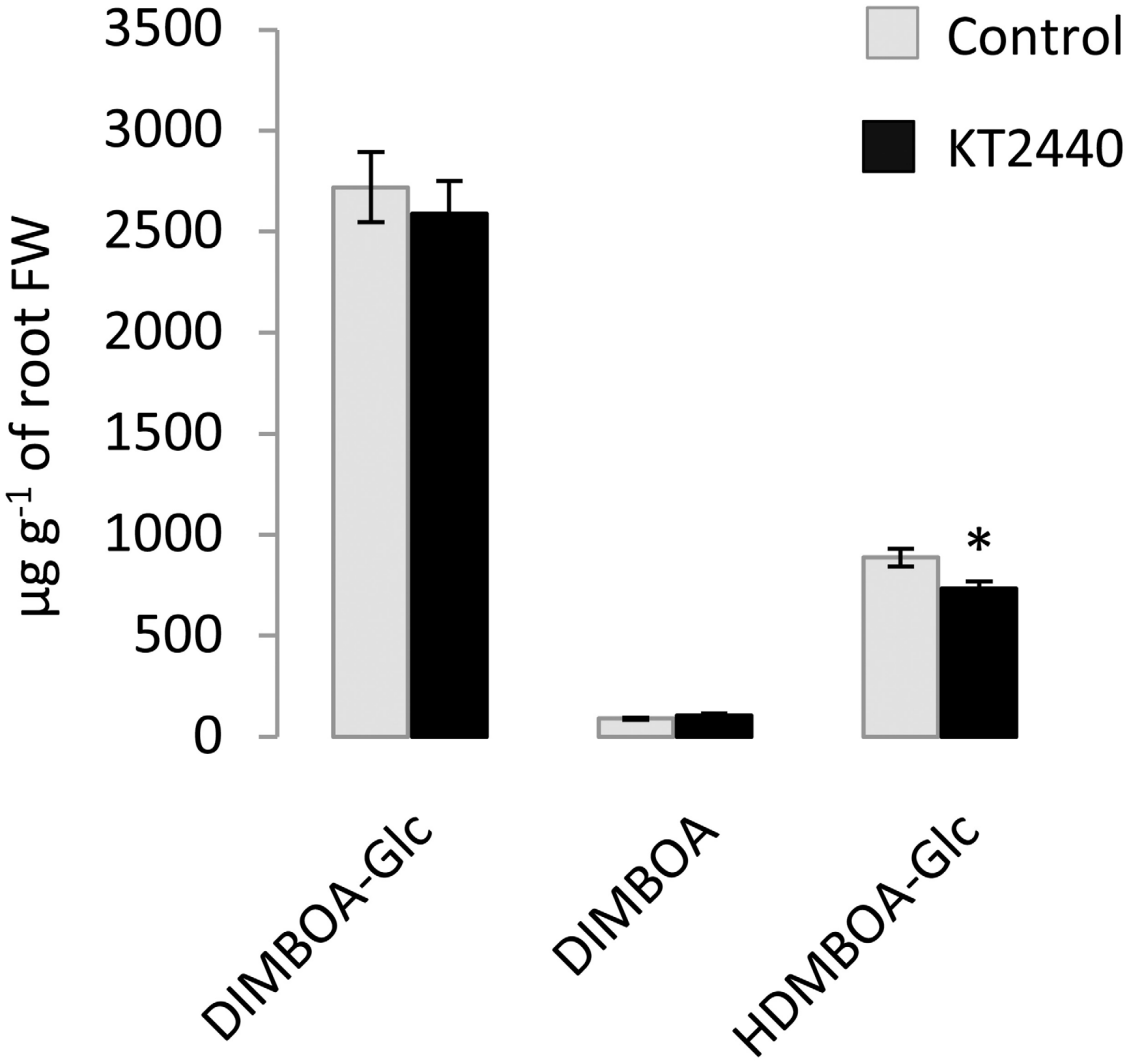
**Absolute quantification of DIMBOA-Glc, DIMBOA and HDMBOA-Glc (μg g^−1^ of fresh material) in roots 3 days after bacterial or mock inoculation (maize var. Jubilee, *n* = 6 replicates, each replicate representing a pool of three plants; plant material is coming from two independent experiments)**. Star indicates a significant difference between control and bacteria-inoculated plants calculated by using a *t*-test: ^*^ if *p* < 0.05.

Several phospholipids were found to be more abundant in KT2440-inoculated plants compared to control plants (Table 1 and Table S3). The most representative marker of KT2440-inoculated plants (i.e., the loading contributing most to the bacteria-inoculated group according to PC1, see Table S3) had a mass-to-charge ratio (*m/z*) of 782.5686 (C_44_H_81_NO_8_P; error 1.8 ppm). This compound was significantly 14-fold more abundant in bacterized roots than in controls (*p* = 0.002; Mann–Whitney Rank Sum Test). This phospholipid was identified as a diacylglycerophosphocholine (a type of phosphatidylcholine) thanks to an MS/MS phosphocholine fragment at *m/z* 184.0741 (C_5_H_15_NO_4_P). As BXD are well-known maize defense compounds, the absolute changes of three BXD were measured in leaves and roots 3 days upon KT2440 inoculation (Figure 3). Changes between control and bacteria-inoculated plants were only observed for HDMBOA-Glc which was slightly but significantly decreased in bacterized roots.

**Table 1:** **Metabolites significantly up- and down-regulated in maize roots upon KT2440 inoculation**.

**Compound**	***m/z***	**RT (min)**	**MF**	**Level of identification**	**PC 1**	**FI**
Diacylglycero-phosphocholine	782.5670	8.34	C_44_H_80_NO_8_P	3	0.598598	14.07^**^
Diacylglycero-phosphocholine	740.5208	8.25	C_41_H_74_NO_8_P	3	0.168817	10.38^**^
Unidentified phospholipid or fragment of phospholipid	714.4706	8.35	C_38_H_68_NO_9_P	3	0.123916	14.51^**^
Unidentified phospholipid or fragment of phospholipid	674.4384	8.35	C_35_H_68_NO_9_P	3	0.110485	7.87^**^
Unidentified phospholipid or fragment of phospholipid	690.4343	8.38	C_35_H_64_NO_10_P	3	0.0851343	22.18^**^
HDMBOA-Glc	388.1247	2.70	C_16_H_21_NO_10_	1	−0.0515908	0.82^*^
Indole-3-butyric acid	204.1038	1.43	C_12_H_13_NO_2_	2b	−0.0516423	0.72^**^
Diacylglycero-phosphocholine	758.5676	8.00	C_42_H_80_NO_8_P	3	−0.0773299	0.76^**^
Unidentified compound	885.5505	9.07	C_54_H_76_O_10_	4	−0.11115	0.01^**^

RT, retention time; MF, molecular formula; PC1, PCA loadings ranking sorted accorded to first component; FI, fold induction (bacterized vs. control plants). Asterisks indicate p value (bacterized vs. control plants, Mann–Whitney Rank Sum Test;

* p < 0.05,

** *p < 0.01). Levels of identification were determined according to Schymanski et al. (2014)*.

**Figure 4:**
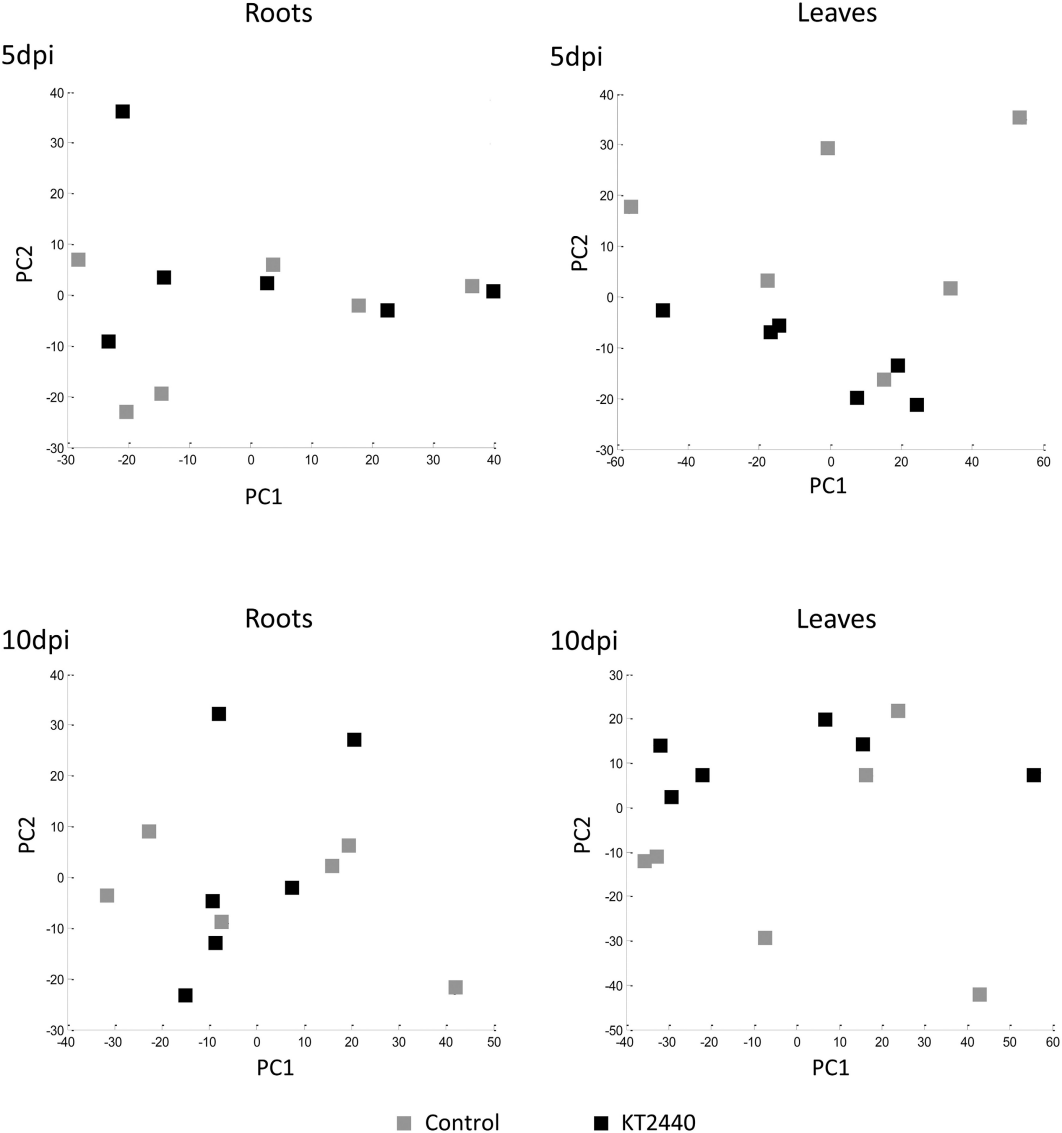
**Principal component analysis (PCA) score plot of metabolomic fingerprinting of maize roots and leaves (var. Jubilee) 5 or 10 days after treatments (*n* = 6 replicates, each replicate representing a pool of 3 plants)**. Gray squares represent non-bacterized plants, black squares represent bacterized plants.

PCA performed at other time-points (5 and 10 dpi) did not separate the treatments (Figure 4 and Table S4), showing no significant changes in secondary metabolite profiles in roots and in leaves later in KT2440-plant interaction, already 5 days after KT2440 inoculation.

### Induced systemic resistance (ISR) against *C. graminicola*

**Figure 5:**
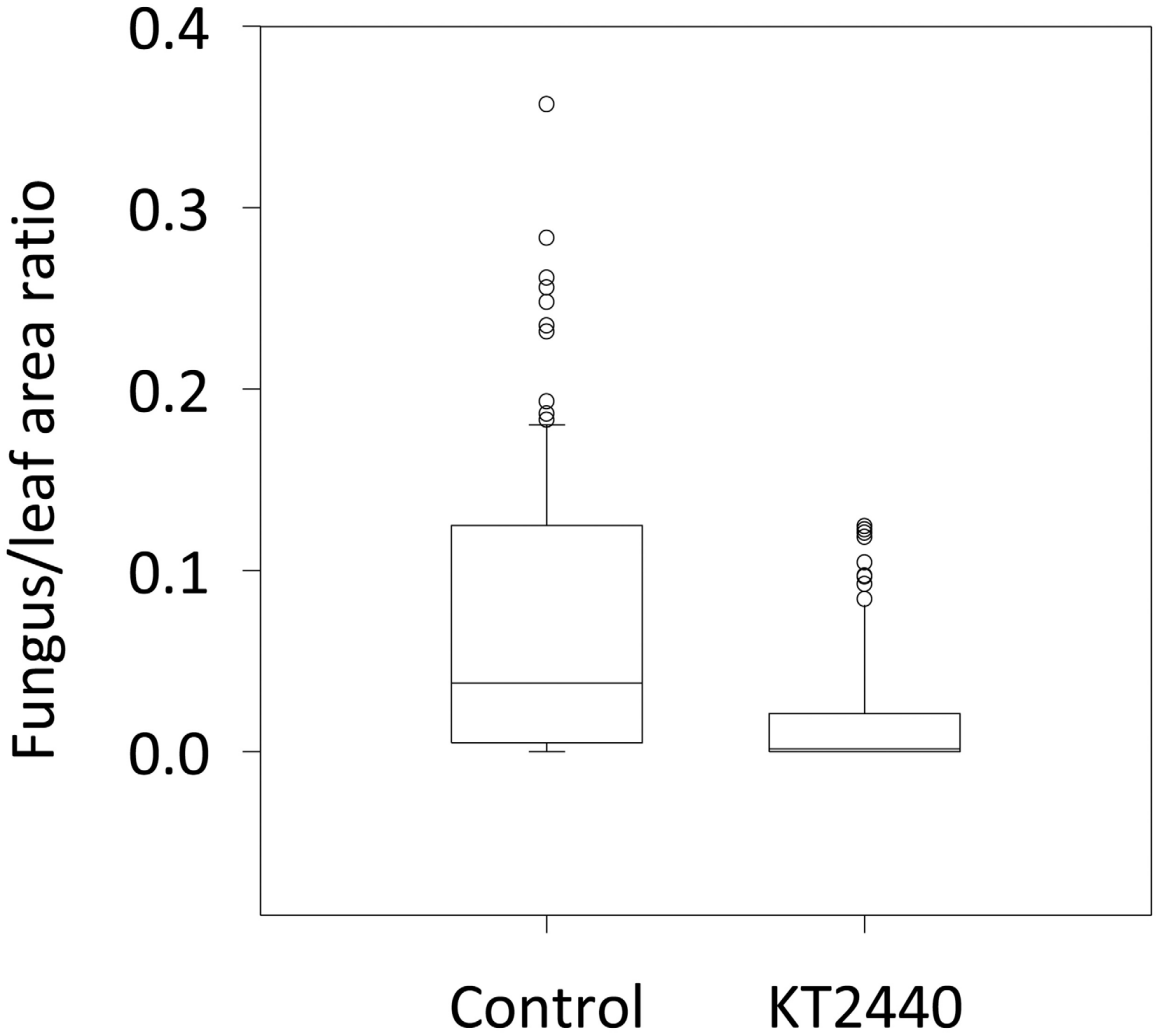
**Corn anthracnose severity for mock-treated (control) and KT2440-inoculated plants**. Twelve-day old maize plants var. Jubilee were inoculated with a spore suspension of *C. graminicola*. Fungal growth in mock-treated (control) and KT2440-inoculated plants was measured 4 days after infection (during the necrotrophic phase) by calculating the ratio between fungal and leaf areas on a predetermined leaf area (*n* = 24 plants per treatment, from two independent experiments). Treatments were statistically different (*p* < 0.001; Mann–Whitney Rank Sum test). Dots represent outliers.

**Figure 6:**
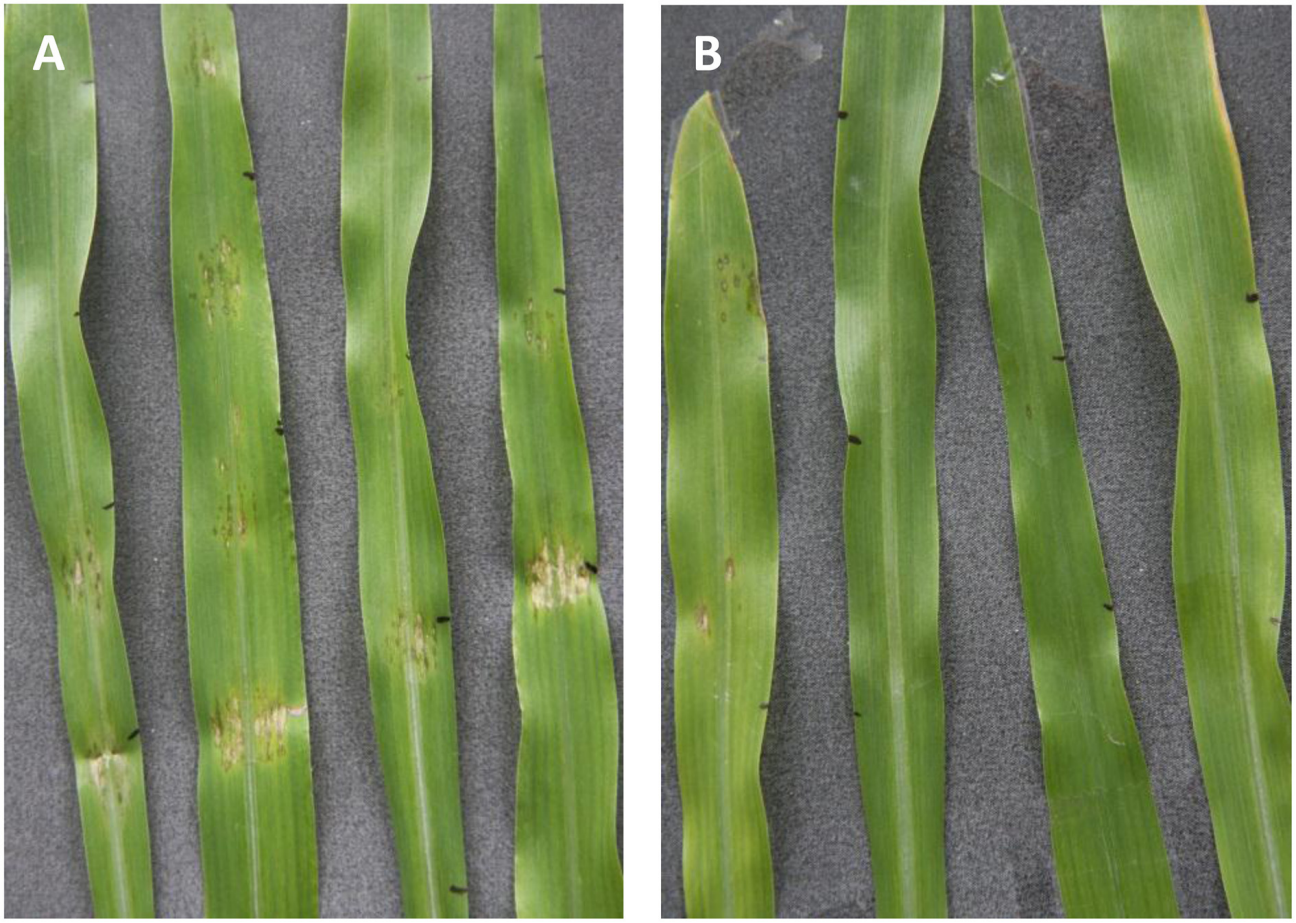
**Symptoms on maize leaves (var. Jubilee) due to infection by *C. graminicola* on mock-treated plants **(A)** and KT2440-inoculated plants **(B)** 4 days after inoculation (corresponding to the necrotrophic phase)**. Lesions are almost absent for KT2440-inoculated plants.

To test the resistance induced by KT2440 against *C. graminicola* in maize leaves, fungal colonization of the tissue was measured 4 days after infection (dai), corresponding to 2 days after the appearance of leaf necrosis. The leaf area colonized by *C. graminicola* was 20 times smaller in bacterized plants compared to non-bacterized plants (*p* < 0.001, Mann–Whitney Rank Sum test; Figure 5). These differences are clearly visible in Figure 6. Mock-treated infected plants show necrosis at infection sites (marked by a black dot), whereas KT2440-inoculated plants show almost no necrosis.

Four days after inoculation with *C. graminicola* bacterial presence in maize roots and leaves was quantified. No KT2440 were present in leaves of control and KT2440-inoculated plants, showing that at this plant growth stage, KT2440 do not move in the upper plant parts. Whereas no KT2440 were found from control roots, 2.97 × 10^6^ CFU g^−1^ of fresh root of KT2440 were counted.

## Discussion

### Root colonization pattern of KT2440 in maize plants

We have shown before that KT2440 cells are not evenly distributed along maize roots: stronger colonization was observed in basal root parts in comparison to root tips (Planchamp et al., 2013). Here, we investigated maize root colonization by KT2440 during plant growth and ISR. Despite a decreased concentration of KT2440 during plant growth, in all the experiments, KT2440 cell numbers reached at least 10^5^ CFU g^−1^ of fresh root 10 dpi and 4 days after *C. graminicola* inoculation. This concentration corresponds to a normal bacterial colonization of the rhizoplane that ranges from 10^5^ to 10^7^ CFU g^−1^ of fresh root weight (Benizri et al., 2001). Considering that KT2440 is not affected by our growing system (Planchamp et al., 2013) and is a competitive strain under non-sterile soil conditions (Neal et al., 2012), the decreasing number of KT2440 cells is most probably due to normal colonization stabilization instead of potential competition or non-adequate external conditions. This study on KT2440 root colonization pattern confirmed the efficiency of KT2440 to colonize roots of maize var. Jubilee and its fluctuation during plant growth before reaching a stable colonization.

### Possible plant defense manipulation during maize root-KT2440 interaction

To investigate local and systemic plant responses upon KT2440 inoculation, transcriptional analysis of stress- and defense-related genes and metabolite profiling were carried out. Only few transcriptional changes and weak transcript responses were observed. This observation is in agreement with other similar studies involving rhizobacteria-triggered ISR in which the capacity of the rhizobacteria to induce plant resistance against attacks was not associated with extended transcriptional changes but with a primed plant response to attacks (Van Wees et al., 2008). Most changes occurred in roots 3 days after KT2440 inoculation. Changes were different in roots and in shoots suggesting distinct local and systemic responses to KT2440 root colonization. Some ET- and auxin-related genes were regulated in leaf tissues but not in roots while some ABA- and SA-dependent genes were regulated in roots but not in leaves.

Our transcriptional analysis (Figure 1) revealed that in contrast to Arabidopsis response to *P. fluorescens* GM30 (Weston et al., 2012) and WCS417 (Van Wees et al., 2008), no ET response was induced in maize roots upon KT2440 inoculation. However, some JA- and ABA-related genes were up-regulated early during colonization with KT2440. Interestingly, some SA-related genes were down-regulated later in the interaction. Attenuation of SA signaling via hormonal crosstalk mechanisms has been shown for a wide variety of beneficial microbes such as PGPF (Jacobs et al., 2011), mycorrhiza, and rhizobia (Gutjahr and Paszkowski, 2009) and also PGPR (Okubara et al., 2010). This mechanism helps plant colonization by beneficial organisms. It is tempting to hypothesize that KT2440 exploit hormone-regulated defenses to establish an efficient colonization by diminishing SA-triggered resistance of maize roots. NCED, an ABA-related gene, was locally up-regulated in maize 3 days after KT2440 inoculation. Interestingly, inoculation with *P. fluorescens* GM30 induced ABA-signaling 3 days after inoculation, whereas *P. fluorescens* Pf-5 did not, indicating a strain-dependent response of Arabidopsis roots (Weston et al., 2012). ABA has a well-known role in plant defense against abiotic stresses but recent studies showed also its involvement in plant hormone regulation (Pieterse et al., 2012) and as potential systemic signal within plants (Balmer et al., 2013). Therefore, the ABA-dependent pathway could play a role in a hormonal crosstalk and MTI suppression or in a root to shoot signaling during KT2440-maize interaction, or both. Further studies are planned to confirm these hypotheses.

Several interactions between beneficial microbes and plants have been associated with BXD ratio changes that were microbe strain and plant cultivar dependent. Song et al. (2011) showed for example that the presence of *G. mosseae* in maize roots induced an increase of DIMBOA in roots. In response to inoculation with *Azospirillum* strains, DIMBOA content varied in a strain-dependent manner (increase or decrease) or did not vary compared to control (Walker et al., 2011). *P. fluorescens* F113, *A. lipoferum* CRT1, and *G. intraradices* JJ291 had a differential effect on BXD content on inoculated plants (Walker et al., 2012). Considering that KT2440 use DIMBOA exudated from maize roots as a chemotaxis signal and that KT2440 are tolerant to this biocidal compound (Neal et al., 2012), we initially hypothesized that KT2440 could manipulate BXD production in maize roots by increasing DIMBOA content in order to control rhizoplane colonization by competing rhizobacteria that would be less tolerant to the presence of DIMBOA. In this study, we found an up-regulation of *Bx1* in roots of KT2440-inoculated plants that could indicate an enhanced BXD production due to the plant-bacteria interaction. However, despite the up-regulation of BXD-dependent pathway, no significant increase of BXD was found in bacterized roots. Furthermore, HDMBOA-Glc was reduced in KT2440-inoculated roots at 3 dpi (Figure 3). As the concentration of BXD in roots does not necessarily correlate with concentration in root exudates (Niemeyer, 2009), BXD could have been present out of roots into roots exudates Even if further investigations would be necessary, our results tend to show a limited importance of BXD in the interaction between KT2440 and maize roots.

### Importance of phospholipids in KT2440-maize interaction

In the course of our metabolite profiling (Table 1, Figures 2, 4), phospholipids were found to be the most significantly altered metabolites in roots 3 days after KT2440 inoculation. Phospholipids are structural components of biological membranes that also play a key role during perception of extracellular signals that are necessary during plant-microbe interactions. In *Brassica napus*, the involvement of phospholipases in early response to systemic acquired resistance and induced systemic resistance inducers was demonstrated (Profotova et al., 2006). As reviewed by Cowan (2006), phospholipid-dependent mechanisms are linked to the mechanism of action of plant hormones involved in defense responses, such as ABA, ET, auxin, JA, and cytokinin.

Phosphatidylcholine (PC), the most significantly induced compound found in KT2440-inoculated plants as compared to control plants (Table 1), is at the origin of production of important defense molecules. Hydrolyzation of PC by phospholipase A results in the production of fatty acids and lysophosphatidylcholine (LPC). Fatty acids are linked to JA-related pathway through their role in oxylipin and jasmonate biosynthesis. These defense compounds are generated via the activity of lipoxygenase (LOX). Interestingly, in our study *LOX3* was up-regulated concomitantly with an increased PC production, suggesting a possible role of phospholipids in the maize-KT2440 interaction through JA-related pathway activation. Moreover, recently it has been shown that plant lipid derivatives are key elements in cell–cell communication for KT2440. Fatty acids activate a KT2440 gene that is important for population density control (Fernández-Piñar et al., 2012). Moreover, Drissner et al. (2007) proposed LPC as an important messenger that can transduce early signals during potato root colonization by mycorrhiza. The present study underlines the potential role of lipid derivatives during maize-KT2440 efficient colonization through an effect on plant immunity and bacterial density regulation. It would be interesting to further elucidate the role of phospholipids in maize-KT2440 early interactions because, as for mycorrhizal interaction, phospholipids could play a role as messenger in signal transduction following KT2440 colonization (Drissner et al., 2007).

### ISR triggered by KT2440 against *C. graminicola*

Necrotrophic but also hemibiotrophic pathogens are negatively affected by plant defenses induced by beneficial microbes. For example, rice resistance triggered by mycorrhiza or rhizobacteria was phenotypically associated with a reduction of lesions caused by *M. oryzae* (De Vleesschauwer et al., 2008, 2009; Campos-Soriano et al., 2012). *C. graminicola*, the causal agent of maize anthracnose, has a worldwide impact on maize production and leads to important economic losses (Frey et al., 2011). This fungal pathogen has a hemibiotrophic life-style that starts with a biotrophic phase and then switches to a predominantly necrotrophic phase in which only the hyphal tips remain biotrophic. The transition to partial necrotrophy is phenotypically characterized by visible leaf necrosis around 2–3 days after fungal leaf inoculation in laboratory conditions (Vargas et al., 2012; Balmer et al., 2013). In our study, bacterized plants did not exhibit (or only to a small extend) fungal growth and leaf necrosis caused by *C. graminicola* inoculation in contrast to non-bacterized plants (Figures 5, 6) where necroses were observed already after 2–3 days as described in Balmer et al. (2013) and were well developed after 4 days. This points to a bioprotective effect of KT2440 that inhibits fungal growth early in the interaction. Given that no KT2440 were found in leaves and that no pronounced changes in known defense secondary metabolites (e.g., BXD) were observed in maize leaves after KT2440 treatment, it strongly suggests that the possibility of a bacterial effect against *C. graminicola* via a direct antagonism is unlikely. Coupling this result and phenotypic observations points to a possible priming of plant defenses instead of a direct induction of different defense mechanisms by KT2440. ISR mechanisms are known to be effective against a wide range of attacks. In a preliminary study (data not shown), we found that the presence of KT2440 in maize roots negatively affected larval weight of the generalist herbivore *Spodoptera littoralis* that resulted from the decreased quantity of leaf material consumed. On the contrary, the larvae of the specialist *Spodoptera frugiperda* were not affected by the presence of KT2440. Hence, it seems that KT2440 has a potential as an ISR-inducer also against leaf chewing herbivores in maize plants and that this resistance seems to depend on the degree of host specialization.

KT2440 have been studied for their capacity to closely interact with maize plants by adhering efficiently to maize seeds and for being attracted by maize plants that release DIMBOA into the rhizosphere (Espinosa-Urgel and Ramos, 2004; Neal et al., 2012). However, little information is available concerning their direct effect on plant physiology and their potential as defense inducers in maize plants. Here, we showed that efficient root colonization with KT2440 was accompanied by a plant reaction at the transcriptomic and metabolomic level, mainly early during the interaction. These changes corroborated our hypothesis that some plant defense mechanisms are activated in response to KT2440 inoculation, as the plant seems to firstly perceive the bacterial presence as a threat. Later in the interaction a sort of accommodation state of the plant to the presence of KT2440 seems to set in, visible as an attenuated plant response. Considering that KT2440 are rhizobacteria restricted to the rhizoplane and without an endophytic colonization (Wu et al., 2010), it could explain the weaker and shorter-lasting changes observed in plants colonized by KT2440 compared to more intricate beneficial interactions involving plants and mycorrhiza, rhizobia, or endophytic bacteria.

This study also demonstrates the ability of KT2440 to induce maize systemic resistance against *C. graminicola* and preliminary experiments tend to indicate that KT2440 could induce maize resistance against additional biotic stresses such as herbivory. Our study shows the possibility of KT2440 being interesting rhizobacteria for agricultural use. Understanding of defense mechanisms involved in systemic resistance induced by KT2440 in maize could be of great importance for the development of future crop protection methods by exploiting root defense induction via the use of beneficial microbes.

### Conflict of interest statement

The reviewer Victor Flors declares that, despite having collaborated with authors Gaetan Glauser and Brigitte Mauch-Mani, the review process was handled objectively and no conflict of interest exists. The authors declare that the research was conducted in the absence of any commercial or financial relationships that could be construed as a potential conflict of interest.
